# Collaborating single-cell and bulk RNA sequencing for comprehensive characterization of the intratumor heterogeneity and prognostic model development for bladder cancer

**DOI:** 10.18632/aging.205166

**Published:** 2023-11-06

**Authors:** Jie Wang, Zili Zuo, Zongze Yu, Zhigui Chen, Lisa Jia Tran, Jing Zhang, Jinsong Ao, Fangdie Ye, Zhou Sun

**Affiliations:** 1 Department of Urology, The Second People’s Hospital of Meishan, Meishan, Sichuan 620500, China; 2 Department of Urology, The First People’s Hospital of Jiangxia, Wuhan 430200, Hubei, China; 3 Department of Urology, China-Japan Union Hospital of Jilin University, Changchun 130000, Jilin, China; 4 Department of General, Visceral, And Transplant Surgery, Ludwig-Maximilians-University Munich, Bayern, Munich 81377, Germany; 5 Division of Basic Biomedical Sciences, The University of South Dakota Sanford School of Medicine, Vermillion, SD 57069, USA; 6 Department of Urology, Huashan Hospital, Fudan University, Jing’an 200000, Shanghai, China

**Keywords:** bladder cancer, tumor heterogeneity, prognostic model, single-cell RNA sequencing, cancer-associated fibroblasts

## Abstract

Introduction: Gaining a deeper insight into the single-cell RNA sequencing (scRNA-seq) results of bladder cancer (BLCA) provides a transcriptomic profiling of individual cancer cells, which may disclose the molecular mechanisms involved in BLCA carcinogenesis.

Methods: scRNA data were obtained from GSE169379 dataset. We used the InferCNV software to determine the copy number variant (CNV) with normal epithelial cells serving as the reference, and performed the pseudo-timing analysis on subsets of epithelial cell using Monocle3 software. Transcription factor analysis was conducted using the Dorothea software. Intercellular communication analysis was performed using the Liana software. Cox analysis and LASSO regression were applied to establish a prognostic model.

Results: We investigated the heterogeneity of tumors in four distinct cell types of BLCA cancer, namely immune cells, endothelial cells, epithelial cells, and fibroblasts. We evaluated the transcription factor activity of different immune cells in BLCA and identified significant enrichment of TCF7 and TBX21 in CD8+ T cells. Additionally, we identified two distinct subtypes of cancer-associated fibroblasts (CAFs), namely iCAFs and myoCAFs, which exhibited distinct communication patterns. Using sub-cluster and cell trajectory analyses, we identified different states of normal-to-malignant cell transformation in epithelial cells. TF analysis further revealed high activation of MYC and SOX2 in tumor cells. Finally, we identified five model genes (SLCO3A1, ANXA1, TENM3, EHBP1, LSAMP) for the development of a prognostic model, which demonstrated high effectiveness in stratifying patients across seven different cohorts.

Conclusions: We have developed a prognostic model that has demonstrated significant efficacy in stratifying patients with BLCA.

## INTRODUCTION

Bladder cancer (BLCA) emerges as a leading one of the most commonly diagnosed solid tumors and accounts for the second most common malignant tumor in the genitourinary tract, with an incidence rate of over 400,000 new cases per year and currently affecting over two million patients [1, 2]. Recent efforts in cancer prevention and treatment have resulted in a substantial decrease in mortality rates of BLCA over the past few decades, especially in developing countries such as China [3, 4]. Nevertheless, patients with BLCA face a high risk of cancer metastasis, and the prognosis for metastatic BLCA remains poor [5]. Consequently, early diagnosis and treatment are critical components of effective BLCA management.

Transcriptomics has emerged as a valuable tool for analyzing the gene expression signatures of various types of cancer. The Cancer Genome Atlas (TCGA) has demonstrated the benefits of utilizing gene expression signatures and clinical features to classify patients based on their treatment responses and disease outcomes. Previous literature has focused on finding molecular markers of disease [6–8]. However, resolving intratumoral heterogeneity remains a significant challenge, leading to expression profiles that primarily represent the “average” molecular characteristics of highly heterogeneous cancer cells [7, 8]. scRNA-seq has emerged as a powerful tool for transcriptomic profiling of individual cancer cells, facilitating the clinical application of more personalized treatments [9]. Deeper insight into the scRNA-seq results of BLCA is crucial for understanding the mechanisms of BLCA carcinogenesis and progression [10, 11].

In this study, we investigated the heterogeneity of tumor cells in four distinct cell types of BLCA: immune cells, endothelial cells, epithelial cells, and fibroblasts. We evaluated the transcription factor (TF) activity of different immune cells in BLCA and identified significant enrichment of TCF7 and TBX21 in CD8+ T cells. Additionally, we identified two distinct subtypes of cancer-associated fibroblasts (CAFs), namely iCAFs and myoCAFs, that exhibited distinct communication patterns. Using sub-cluster and cell trajectory analyses, we identified different states of normal-to-malignant cell transformation in epithelial cells. TF analysis further revealed high activation of MYC and SOX2 in tumor cells. Finally, we screened out five model genes (SLCO3A1, ANXA1, TENM3, EHBP1, LSAMP) for the construction of a prognostic model, which showed high effectiveness in stratifying patients across seven different cohorts. Collectively, our findings offer new insights into potential precision immune treatments for BLCA.

## MATERIALS AND METHODS

**Figure 1:**
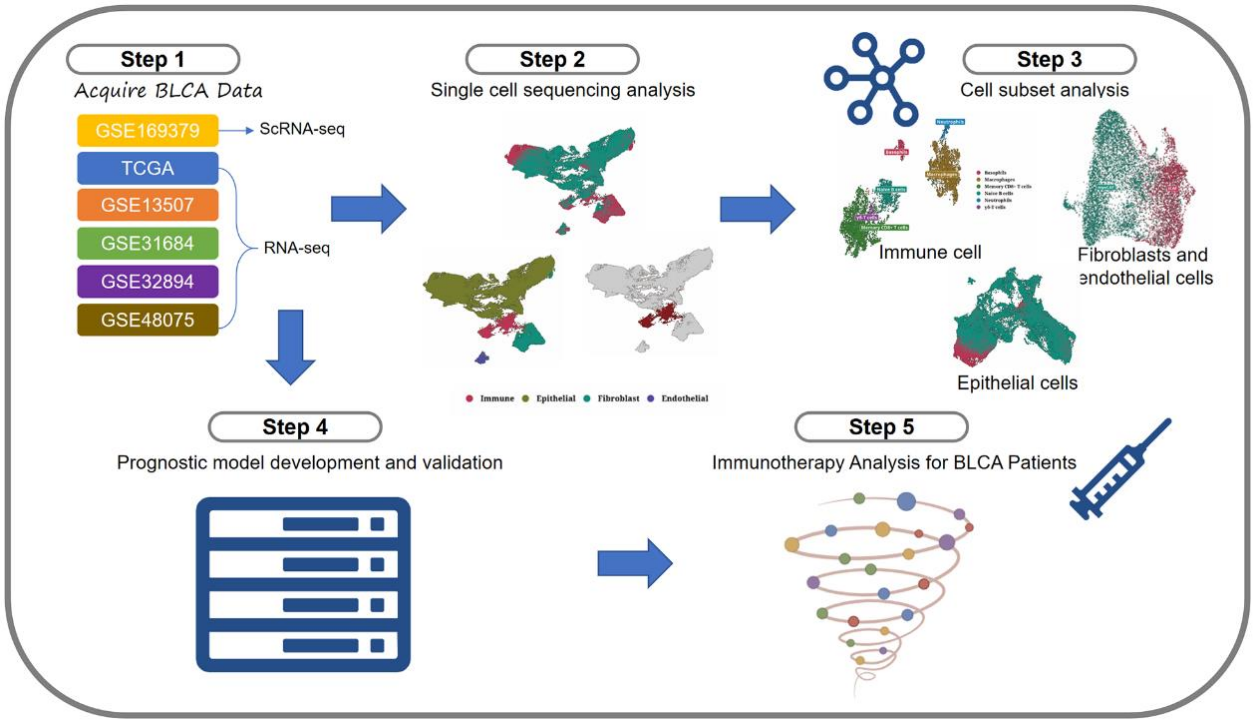
Flowchart.

The flowchart was shown in Figure 1.

### Procurement and processing of transcriptome data

The RNA expression profiles and corresponding clinical data of bladder cancer patients (n = 399) were obtained from the TCGA-BLCA cohort of the TCGA database (http://cancergenome.nih.gov/). Samples with a survival time of less than 30 days were excluded. The remaining samples were randomly divided into a training group and a validation group in a 5:5 ratio. All data were normalized to FPKM format and then converted to log2 format for subsequent analysis. Additionally, to validate the model, the microarray data from GSE13507, GSE31684, GSE32894, and GSE48075 in the Gene Expression Omnibus (GEO) database (http://www.ncbi.nlm.nih.gov/geo/) were used as a validation set [12–15]. The batch effect was eliminated using the “removeBatchEffect” function in the “limma” package. Furthermore, the IMvigor210 dataset was utilized for the analysis of immunotherapy.

### Acquisition and processing of scRNA-seq data

The scRNA-seq dataset utilized in this study was obtained from GSE169379 [16]. The tumor samples from 25 patients and adjacent samples from four patients were included. Data analysis was performed using the Seurat package. The h5ad file, which was processed in the original literature, was directly downloaded for cytoplasmic control. Highly variable genes were identified and corrected using the SCTransform method, while the batch effect was addressed by the Harmony approach. Subsequently, dimensionality reduction methods such as Uniform Manifold Approximation and Projection (UMAP), t-distributed Stochastic Neighbor Embedding (t-SNE), and the Louvian clustering algorithm, all from Seurat, were employed. Differentially expressed genes (DEGs) analysis between clusters or cell types was performed using the FindAllMarkers function with the parameters set at a p-value less than 0.05, an absolute value of the log2 fold change greater than 0.25, and an expression ratio greater than 0.1.

### Cell annotation analysis

In order to distinguish immune cells from other cell types, PTPRC (CD45) was employed as the marker. Subsequently, markers for epithelial cells (EPCAM, KRT18, KRT19, GRHL2), fibroblasts (COL1A1, COL1A2, COL3A1, ACTA2), and endothelial cells (PECAM1, CD34, CDH5, VWF) were used to distinguish the remaining cells. Based on these markers, epithelial cells were separated and clustered separately to investigate their heterogeneity within the tumor. Various visualization techniques, including UMAP, t-SNE, histograms, and heat maps, were employed to explore the cellular landscape.

### Subgroup analysis of specific cell group

In order to perform a subgroup analysis of each cell group, immune cells, epithelial cells, fibroblasts, and endothelial cells were separated and further distinguished using the standard Seurat pipeline. Specific markers and sctype software were employed as the basis for grouping, and UMAP diagrams were generated for visualization.

### CNV analysis of epithelial cells

To analyze CNV in the epithelial cells, we utilized the InferCNV software with normal epithelial cells serving as the reference. The primary objective was to identify malignant cells within the subsets of tumor cells.

### Pseudo-timing analysis of epithelial cells

We performed pseudo-timing analysis on the subsets of epithelial cells using the monocle3 software with default parameters, which involved inferring developmental trajectories and ordering of cells based on transcriptomic changes.

### Analysis of transcription factors and tumor-related pathways

To perform transcription factor analysis of each cell subgroup, we utilized the Dorothea software to calculate activity scores at a cellular basis, which describe the enrichment of transcription factors and their downstream targets (regulators) in each cell.

### Analysis of intercellular communication

Intercellular communication analysis was performed using the Liana software. The algorithm utilized a combination of techniques, including “natmi,” “connectome,” “logfc,” “sca,” and “cellphonedb,” to analyze the communication between cells of each cell type.

### Establishment of a prognostic model

To establish tumor-related risk characteristics, we first employed univariate Cox analysis to identify tumor-related genes with prognostic value. Subsequently, we further screened the genes using LASSO regression to establish a prognostic model [12]. This approach enabled us to generate risk score values for each patient, based on which the patients in the TCGA cohort were divided into high and low risk score value groups. We also examined the correlation between the two sets of predictions and evaluated the accuracy of the model.

### Prediction of immune response

To predict the immune response of BLCA patients in the TCGA database, we utilized the TIDE online website (http://tide.dfci.harvard.edu/) to obtain the TIDE scores of BLCA patients. We input the gene expression files of the TCGA-BLCA and IMvigor210 datasets [17]. After the procedures of standardization and cancer type selection, the TIDE website would return files with TIDE scores of the samples. Based on the TIDE scores, the box plots and histograms were generated to determine the differences in immune response among different groups (patients of high and low risk-score value).

### Correlation analysis of the expression data of the tumor cells

To investigate the correlation between tumor cells and bulk data, we employed the Scissor software to correlate the expression data of TCGA-BLCA with survival data. The alpha value was set to 0.05, and both negative and positive cells related to generation were obtained.

### Single-sample gene set enrichment analysis (ssGSEA)

In this study, we used ssGSEA analysis to calculate the Scissor+ score for each TCGA patient, enabling us to measure the levels of gene set enrichment in each sample [13].

### Statistical analysis

All statistical analyses were conducted using the R language. Cox regression analysis was performed using the R packages “survival” and “survminer” for both univariate and multivariate Cox regression analysis. A significance level of P < 0.05 was used to determine the prognostic significance.

### Data availability statement

All original data can be obtained from the lead author Zhou Sun.

## RESULTS

### Expression profile of BLCA at single-cell resolution

**Figure 2:**
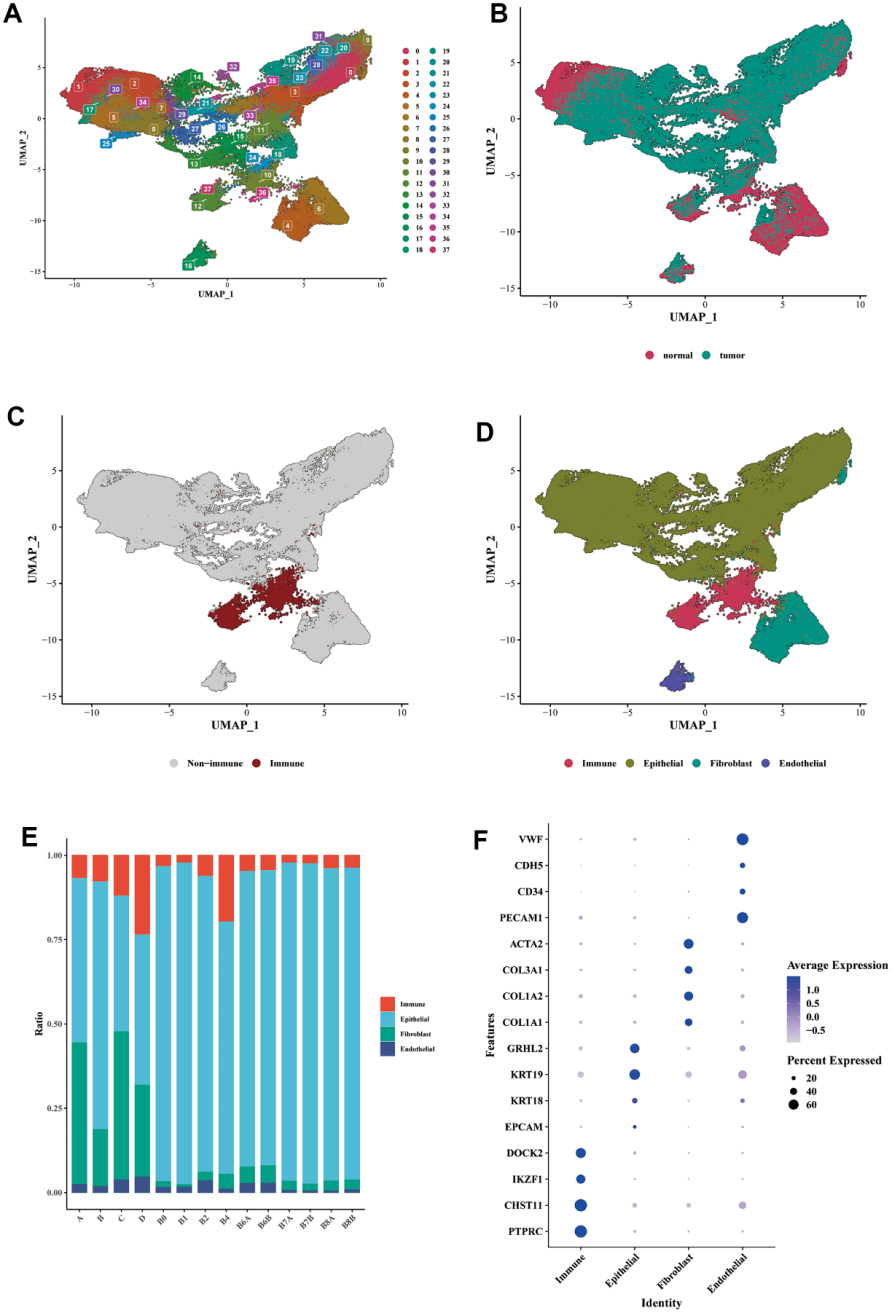
**Single-cell expression profile of BLCA.** (**A**) Dimensionality reduction clustering analysis of the whole cell in the BLCA samples. A total of 38 clusters were separated by different colors. (**B**) UMAP dimensionality reduction diagram showed the distribution of cells from tumor samples and normal samples. (**C**) Annotation of the immune cells cluster using the specific marker of PTPRC (CD45). (**D**) Annotations of epithelial cells, fibroblasts, and endothelial cells were shown in the form of UMAP diagram. (**E**) The proportion of each main cell type in different samples. (**F**) The bubble diagram showed the marker expression of each cell type. Epithelial cell markers: EPCAM, KRT18, KRT19, GRHL2; fibroblast markers: COL1A1, COL1A2, COL3A1, ACTA2; endothelial cell markers: PECAM1, CD34, CDH5, VWF.

After performing dimensionality reduction clustering analysis on the single-cell data, we obtained 38 clusters (Figure 2A). The distribution of cells from tumor samples and normal samples was displayed in the form of a UMAP (Figure 2B). We further utilized the expression of PTPRC (CD45) to determine the immune cell cluster (Figure 2C). Then, epithelial cell markers (EPCAM, KRT18, KRT19, GRHL2), fibroblast markers (COL1A1, COL1A2, COL3A1, ACTA2), and endothelial cell markers (PECAM1, CD34, CDH5, VWF) were used to distinguish the remaining cell types (Figure 2D). Compared to normal tissue samples (A, B, C, D), tumor samples contained a relatively higher proportion of epithelial cells, which could exhibit a malignant phenotype to some extent (Figure 2E). The bubble diagram showed the marker expression of each cell type (Figure 2F).

### Analysis of subclassification of immune cells

**Figure 3:**
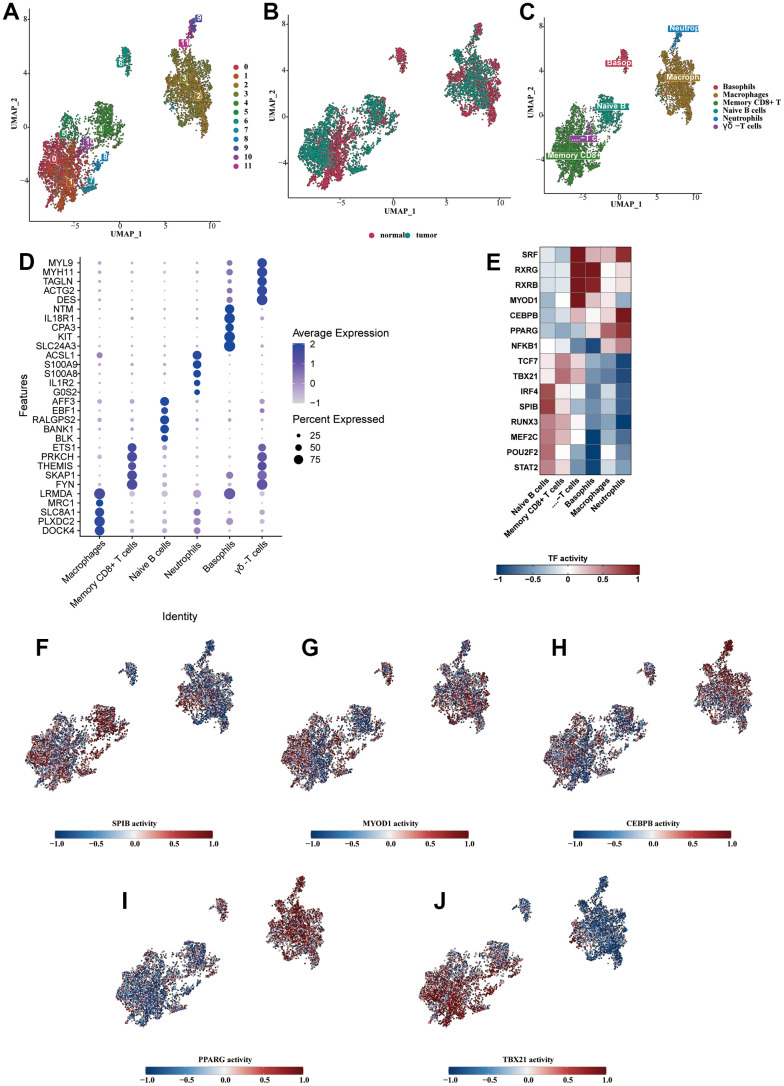
**Analysis of subclassification of immune cells.** (**A**) Further dimensionality reduction clustering analysis of the immune cells. (**B**) The distribution of cells from normal and tumorous tissues in the immune cells. (**C**) Six cell subtypes were obtained by cell annotation of immune cell clusters. (**D**) The bubble diagram showed the specific markers for each cell type. (**E**) The heat map showed the enriched transcription factors of various immune cell types. (**F**–**J**) The highly enriched cytokine activity of each cell type.

The immune cells were isolated and subjected to dimensionality reduction clustering analysis alone. The UMAP generated a total of 12 clusters (Figure 3A). We observed that the sample source of the cells was mainly tumorous samples (Figure 3B). Six cell subtypes were identified through the cell annotation of immune cell clusters (Figure 3C). The bubble diagram showed the specific markers for each cell type, validating the accuracy of the annotation (Figure 3D). We analyzed the transcription factors of various immune cell types and found that B cells were enriched with cytokines such as IRF4, SPIB, RUNX3, MEF2C, POU2F2, and STAT2; CD8+ T cells were enriched with TCF7 and TBX21; γδ-T cells were enriched with SRF, RXRG, RXRB, and MYOD1; basophils were enriched with RXRG and RXRB; macrophages were enriched with PPARG; neutrophils were enriched with cytokines such as SRF, CEBPB, PPARG, and NFKB1 (Figure 3E). We demonstrated the highly enriched cytokine activity of each cell type on the UMAP diagram (Figure 3F–3J).

### Subclassification analysis of fibroblasts and endothelial cells

**Figure 4:**
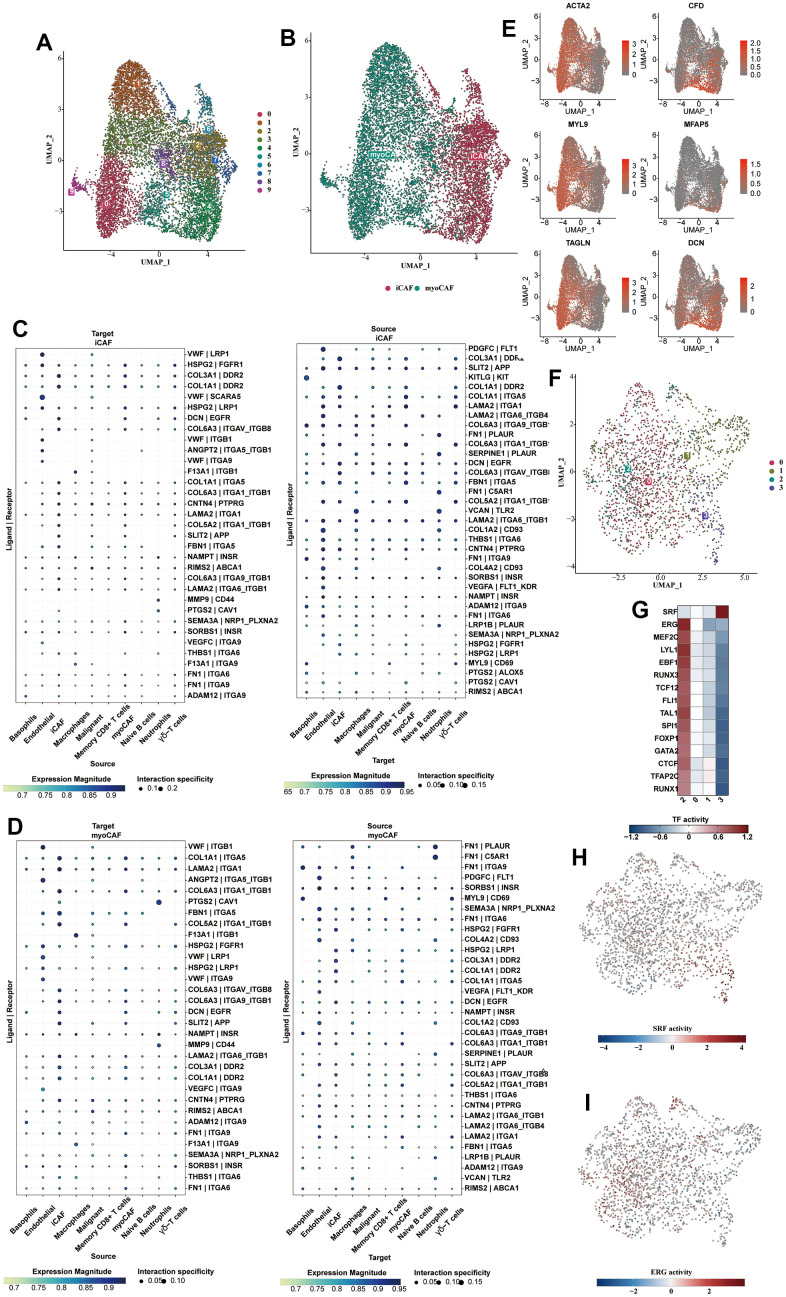
**Subclassification analysis of fibroblasts and endothelial cells.** (**A**) Ten clusters of fibroblasts were shown in the form of a UMAP diagram. (**B**) The distribution of myofibroblasts and inflammatory fibroblasts in the whole fibroblast population. (**C**) The bubble plot showed the cell communication analysis results of iCAFs with different cell types. (**D**) The bubble plot showed the cell communication analysis results of myo-CAFs with different cell types. (**E**) The myofibroblast markers (αSMA, TAGLN, MYLK, MYL9) and iCAFs-specific inflammatory marker genes (CFD, MFAP5, DCN) were shown in the form of bubble map. (**F**) Subclustering of the endothelial cells. (**G**) The heat map showed the enriched transcription factors of various endothelial cell types. (**H**) The transcriptional activity of SRF in different cell types was shown on the UAMP plot. (**I**) The transcriptional activity of ERG in different cell types was shown on the UAMP plot.

Fibroblasts were isolated and subjected to dimensionality reduction cluster analysis. Ten clusters were identified using UMAP (Figure 4A). We divided fibroblasts into myofibroblasts and inflammatory phenotypes, shown on the UMAP plot (Figure 4B). From the cell communication analysis, the ligand receptors used by the two CAFs to communicate with other types of cells were illustrated in Figure 4C, 4D. vWF was highly activated in the iCAFs communication, while FN1 was highly enriched in the myoCAFs. Clusters 0, 1, 3, 5, 8, and 9 were identified as myoCAFs, given the detected up-regulation of myofibroblast markers, including αSMA and contractile proteins (TAGLN, MYLK, MYL9). Clusters 2, 4, 6, and 7 expressed iCAFs-specific inflammatory genes, such as CFD, MFAP5, and DCN (Figure 4E). Four clusters were clustered using UMAP from the endothelial cells (Figure 4F). C2 was enriched in transcription factors such as ERG, and C3 was enriched in SRF (Figure 4G). The transcriptional activity of SRF and ERG in different cell types was shown on the UAMP plot (Figure 4H, 4I).

### Analysis of subclassification of epithelial cells

**Figure 5:**
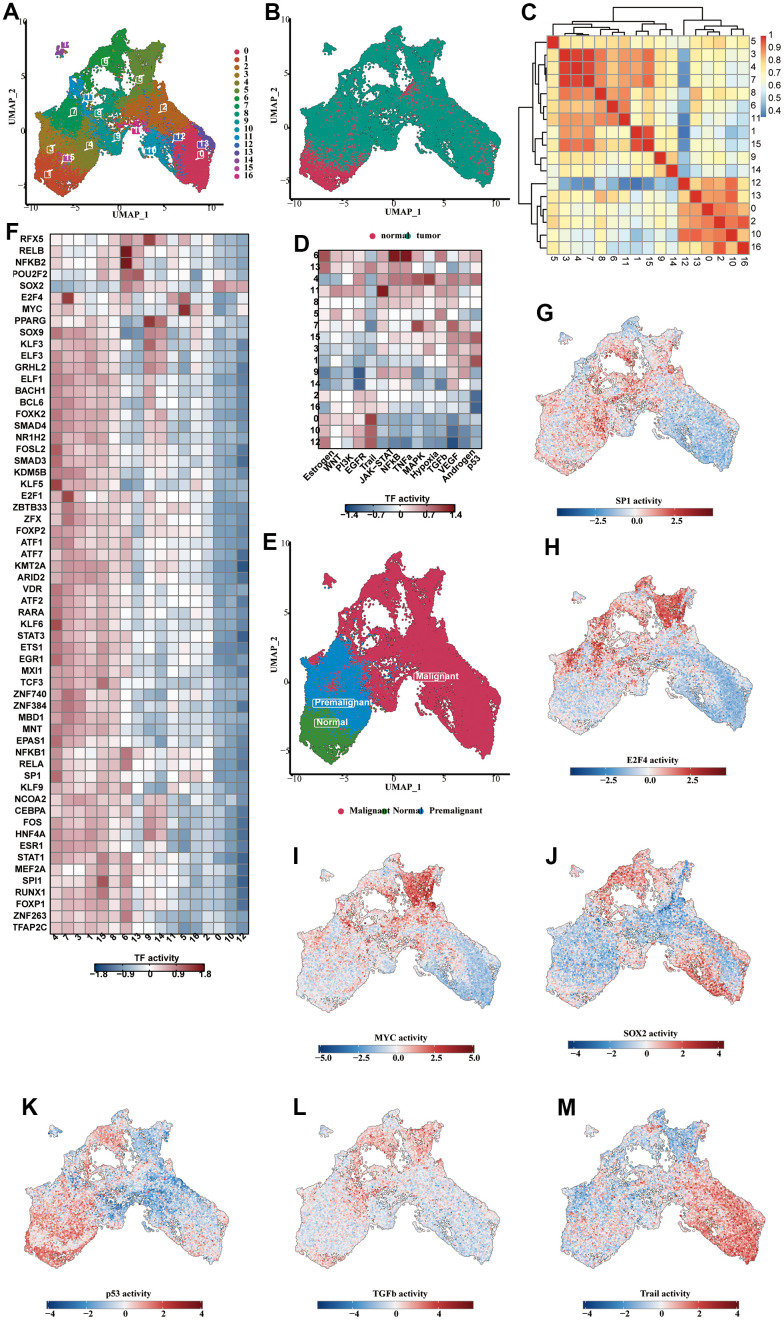
**Analysis of subclassification of epithelial cells.** (**A**) The UMAP diagram showed the 17 subclusters of the epithelial cells. (**B**) The UMAP diagram showed the distribution of normal and tumorous cells in the whole epithelial population. (**C**) The correlation heat map showing the correlative relationship between different subclusters. (**D**) The TF enrichment analysis results, shown in the form of heat map. (**E**) The UMAP diagram showing the distribution of normal, premalignant and malignant cells in the whole cell population. (**F**) The enrichment heat map showing the predicted activity of transcription factors in different subclusters. (**G**–**J**) The predicted activity of SP1 (**G**), E2F4 (**H**), MYC (**I**), SOX2 (**J**) in each of the subclusters, shown in the form of UMAP map. (**K**–**M**) The activity of the dominant functional pathways, including p53 (**K**), NFkB (**L**), Trail (**M**), in each epithelial cell subcluster.

The UMAP diagram showed 17 subclusters of the epithelial cells and the sample type sources of their cells (Figure 5A, 5B). The vast majority of the epithelial cells were derived from the tumorous tissues. We found that c1 and c15 were similar to c3, c4 and c7 on the cluster correlation heat map (Figure 5C). In the pathway analysis, c1 and c15 were highly enriched in the p53 pathway, and c3,4, and 7 were highly enriched in MAPK and hypoxia (Figure 5D). Therefore, c1 and c15 can be identified as normal epithelial cells, while c3, c4 and c7 were intermediate cells experiencing normal-to-tumor transition. c1 and c15 contained a large number of normal epithelial cells, which were consistent with our observation (Figure 5E). We analyzed the enrichment heat map of transcription factors (Figure 5F) and disclosed the activity of dominant transcription factors in each group as a UMAP map (Figure 5G–5I). SP1 was mainly activated in the normal and premalignant cells, while MYC and SOX2 were highly activated in the tumorous cells. The activity of the dominant pathways in each cluster was also shown (Figure 5K–5M). The trail pathway was highly enriched in the malignant cells.

### Cell trajectory and cell communication analysis of epithelial cells

**Figure 6:**
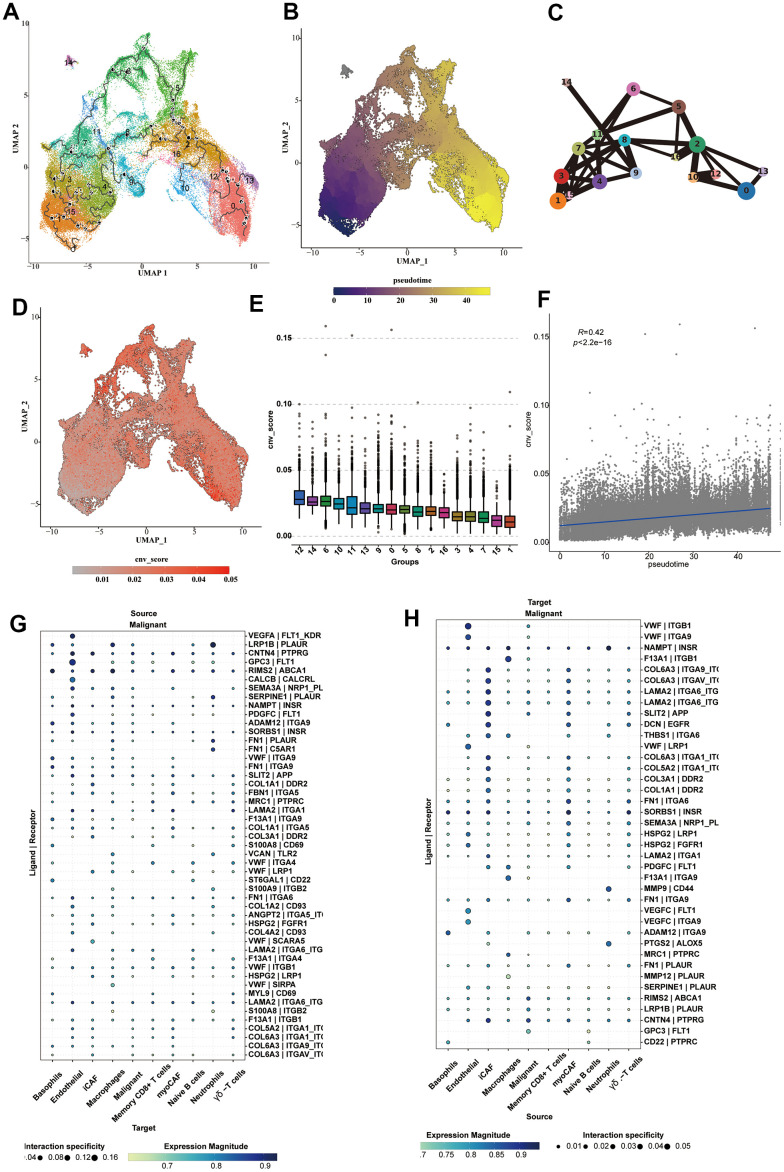
**Cell trajectory and cell communication analysis of epithelial cells.** (**A**) The cell trajectory analysis results of epithelial cells. (**B**) The cell trajectory highlighting the pseudotime results of epithelial cells. (**C**) The cell trajectory analysis results using lines to connect each subcluster, highlighting the transformation status of each cluster. (**D**) The CNV _ score calculated for each epithelial cell, shown on the UMAP diagram. (**E**) The box plot showing the CNV _ score of each cluster. (**F**) The correlation analysis results of CNV _ score and pseudotime. (**G**, **H**) The cell communication analysis on communication between malignant cells and various types of cells.

We analyzed the cell trajectory of epithelial cells and found that normal cells were basically in c1,15, which we confirmed as the starting point of cell trajectory. The inferred cell trajectory started from c1,15, through c3,4,7 (the previously confirmed intermediate state), to the other clusters, which generally belonged to tumor samples (Figure 6A–6C). Moreover, it could be found that the CNV-score also increased with the progression of the above trajectory, which further supported our conclusion (Figure 6D). We also compared the CNV-score of each cluster and found that c1,15 < c3,4,7 < the rest of the subpopulations (Figure 6E). It was also observed that the pseudo-time was positively correlated with the CNV-score (R = 0.42, p < 2.2e-16) (Figure 6F). Subsequently, we performed cell communication analysis on various types of cells, focusing on the communication process between tumor cells and other cell types, and explicitly showing the communication bubble diagrams of tumor cells as source and target, respectively (Figure 6G, 6H). vWF was highly enriched in the endothelial cell to malignant cell communication. An extensive array of ligands was enriched in the cell communication from iCAFs to malignant cells, including LAMA2, COL6A3, SLIT2 and DCN.

### Construction and verification of the prognostic model

**Figure 7:**
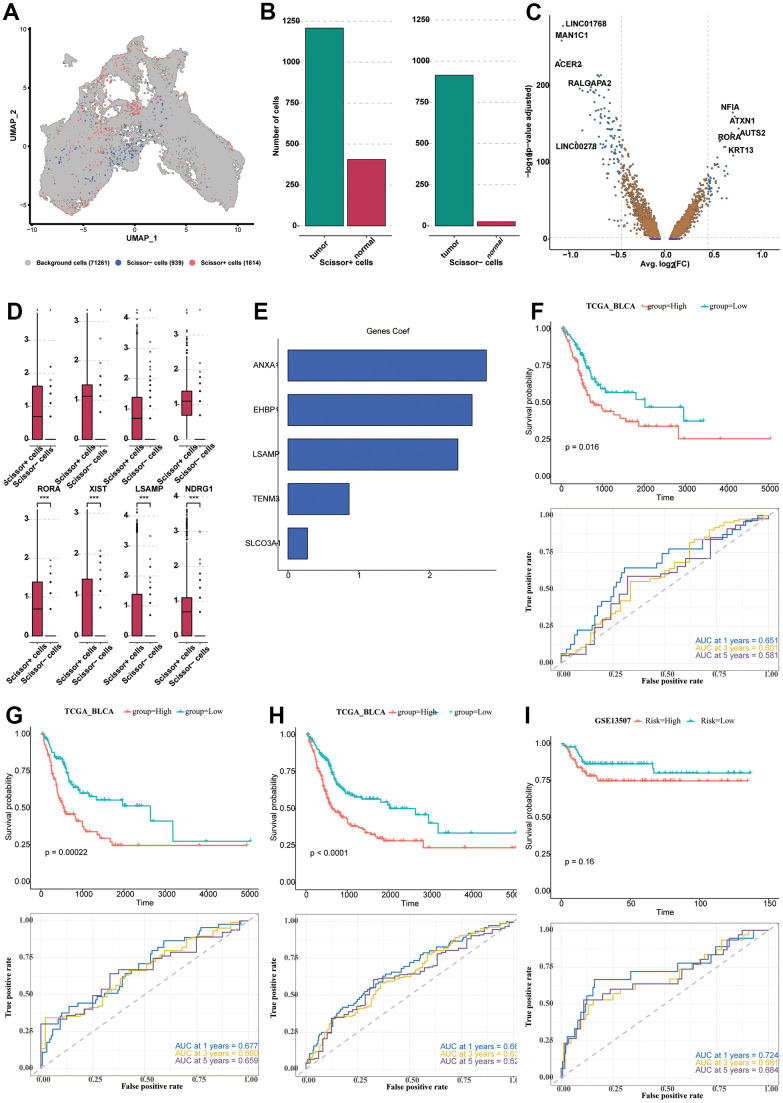
**Construction and verification of the prognostic model.** (**A**) The UMAP diagram showing the classification of positive, negative cells and background cells. (**B**) The column plot showing the composition of positive and negative cells. (**C**) The DEGs analyzed from positive cells and negative cells, shown in the form of the volcano plot. (**D**) The expression levels of the 8 prominent DEGs in the positive and negative cells. (**E**) The five model genes (SLCO3A1, ANXA1, TENM3, EHBP1, LSAMP) and their co-efficient. (**F**–**I**) The survival analysis and ROC curve analysis in all seven utilized datasets. TCGA-BLCA (**F**–**H**); GSE13507 (**I**).

**Figure 8:**
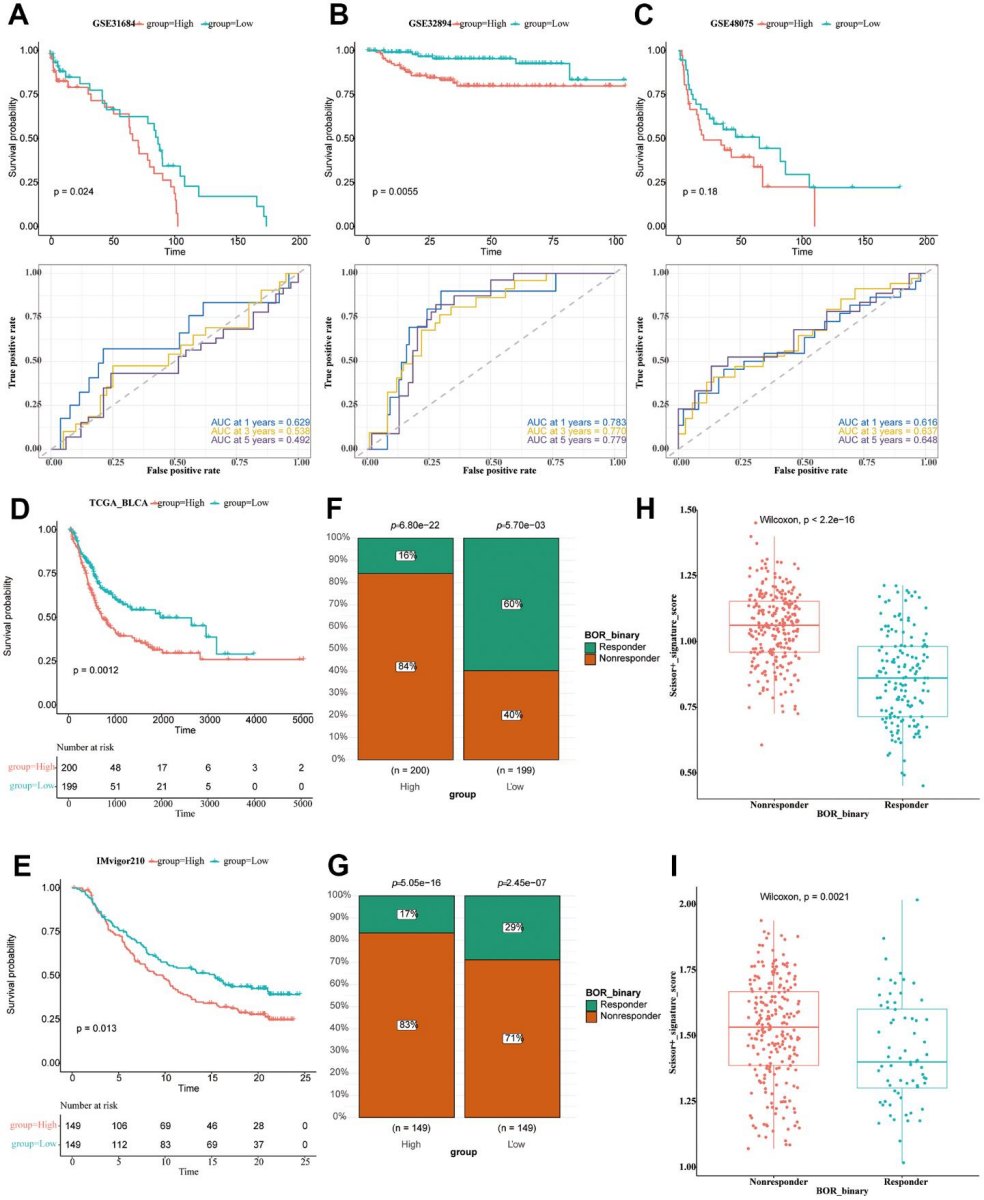
**Immunotherapy analysis of BLCA patients.** (**A**–**C**) The survival analysis and ROC curve analysis in all seven utilized datasets. GSE31684 (**A**); GSE32894 (**B**); GSE48074 (**C**). (**D**) The survival analysis of the TCGA-BLCA patients of high and low riskscore value, shown in the form of KM curve. (**E**) The survival analysis of the IMvigor210 cohort of BLCA patients of high and low riskscore value, shown in the form of KM curve. (**F**) The proportion of responsive and non-responsive patients in different risk groups in TCGA-BLCA patients using the chi-square test. (**G**) The proportion of responsive and non-responsive patients in different risk groups in IMvigor210 cohort using the chi-square test. (**H**) The box plot showing risk value of non-responsive and responsive patients in TCGA-BLCA cohort. (**I**) The box plot showing risk value of non-responsive and responsive patients in IMvigor210 cohort.

We used Scissor software to correlate the expression data of TCGA-BLCA with survival data and single-cell data, and obtained 1614 positive cells, which might exert a relatively greater impact on survival, and 939 negative cells. We used UMAP to classify positive, negative, and background cells (Figure 7A). Then, we analyzed the composition of positive and negative cells. It could be seen that the two groups of cells were both abundant in tumor samples, especially for the negative cells (Figure 7B). The positive and negative cells were analyzed for DEGs, and the results of the difference and distribution were shown in the volcano plot (Figure 7C). We selected 8 highly expressed DEGs in positive cells and plotted the box plot, which showed the expression levels of these DEGs in the positive and negative cells (Figure 7D). In order to further explore the relationship between DEGs and the prognosis of BLCA patients, TCGA-BLCA was used for model construction, and GSE13507, GSE31684, GSE32894, and GSE48075 datasets were used as validation sets. A total of 24 prognostic genes were obtained by univariate Cox analysis (P < 0.05). Next, LASSO and Cox regression analysis were used to develop a prognostic model. Under the optimal regularization parameters, five model genes (SLCO3A1, ANXA1, TENM3, EHBP1, LSAMP) were finally screened out, all of which were risk factors (Figure 7E). We performed ROC curve analysis in all seven utilized datasets to further study the accuracy of differential gene sets in evaluating the prognosis of BLCA patients. The AUC values of the seven datasets were all greater than 0.5 (Figures 7F–7I, 8A–8C). We found that the BLCA of high risk score value had a poor prognosis throughout all seven datasets (P < 0.05).

### Immunotherapy analysis of BLCA patients

We used the 51 obtained DEGs to conduct the ssGSEA analysis to calculate the signature score of each patient as the risk score, which divided the patients into two groups (patients of high and low risk-score value). The immune response of the TCGA sample was obtained by TIDE online analysis. The BLCA patients with high risk-score values in the two data sets (TCGA, IMvigor210) showed an inferior prognosis (Figure 8D, 8E). The chi-square test showed significant differences in the response to immunotherapy (Figure 8F, 8G). In both data sets, we found that the risk value of non-responsive patients was significantly higher than that of the response group (Figure 8H, 8I).

## DISCUSSION

Bladder cancer is one of the most common malignancies of the urinary tract and emerges as one of the most prevalent cancers worldwide [18]. Early diagnosis and treatment are critical components of disease management [8, 19]. In this study, we combined scRNA-seq and bulk RNA-seq to investigate the intratumor heterogeneity of BLCA and developed a prognostic model for BLCA. We investigated the heterogeneity of four cell types of BLCA, evaluated the TF activity of different immune cells in BLCA, identified two distinct subtypes of CAFs, identified different states of normal-to-malignant cell transformation in epithelial cells, and identified five genes to construct a prognostic model.

We comprehensively explored the heterogeneity of four cell types of BLCA, including immune cells, endothelial cells, epithelial cells, and fibroblasts. And we found that tumor samples contained a relatively larger proportion of epithelial cells, which could display malignant phenotype to a certain extent. We identified the malignant state of the epithelial cells in the BLCA samples. Then, we identified different states of normal-to-malignant cell transformation in epithelial cells by sub-cluster and cell trajectory analyses. These subclusters of the malignant phenotype were highly enriched with enhanced MYC TF activity. The canonical role of MYC is to act as a transcriptional regulator in a dimeric DNA-binding complex with MAX [20]. Although MYC-MAX dimers generally stimulate transcription, MYC can also repress target genes via association with the zinc-finger transcription factor Miz-1 [21]. While the number of transcriptionally activated target genes of MYC is substantial, many transcriptionally repressed targets have also been identified. It has been estimated that over 15% of all human genomic loci may be bound and regulated by MYC. MYC is critical in promoting cell-cycle progression via transcriptional activation of genes such as CDK4 and/or cyclin D2 [22, 23]. In addition to this transcriptional function, MYC has also been shown to interact directly with the pre-replicative complex (pre-RC), which controls DNA replication initiation. MYC regulates DNA replication via transcriptional activation of the CDT1 gene that encodes the origin licensing factor Cdt1 [24]. Thus, MYC exerts transcriptional and non-transcriptional control over DNA replication. The regulatory sphere of MYC extends beyond transcriptional regulation as it also controls the expression of miRNAs [25]. MYC activates miR-17-92, a well-characterized miRNA involved in tumorigenesis. The miRNA cluster attenuates the function of E2F transcription factors and has anti-apoptotic effects, indicating that the effect of MYC on cell survival and proliferation is multifaceted [26]. Moreover, MYC plays a critical role in cell death regulation. The overexpression of MYC leads to apoptosis in the absence of survival factors [27]. The induction of apoptosis by MYC depends on the cellular context and can occur through interactions of ARF and MYC, or by the ARF/MDM2/p53 axis [28]. Disturbances in the balance between pro-proliferative and pro-apoptotic functions of MYC can disrupt the ARF/MDM2/p53 pathway or result from cooperative effects of MYC and anti-apoptotic proteins such as BCL2, which can have critical roles in tumorigenesis [29]. In BLCA, the downregulation of PRMT5, another BLCA oncogene, resulted in a significant reduction in the expression of c-Myc, leading to the suppression of cell proliferation and invasion in 5637 and T24 cells, which was mediated through the NF-κB signal pathway. Further clinical endeavours should be dedicated to transforming the molecular outcomes of the muti-functional role of the MYC into clinical trials of practical usage.

Furthermore, we identified two distinct subtypes of CAFs, namely iCAFs and myoCAFs, which exhibited distinct communication patterns. We performed cell communication analysis focusing on the communication process between tumor cells and other cell types. An extensive array of ligands was enriched in the cell communication from iCAFs to malignant cells, including LAMA2, COL6A3, SLIT2, and DCN. It is worth noting that Slit2 was highly enriched in the iCAFs to malignant cell communication. In the breast cancer mouse model, Slit2 was observed to reduce fibrosis by upregulating the expression of matrix metalloproteinase 13 in M1-type tumor associated macrophages (TAMs). Notably, an examination of patient samples demonstrated that high Slit2 expression was significantly associated with improved patient survival and was inversely correlated with the abundance of CD163+ TAMs, indicating that Slit2 might serve as a therapeutic target to reduce fibrosis and prevent the recruitment of pro-tumorigenic TAMs. These evidences suggested Slit2 as an immunotherapeutic agent for reprogramming of TAMs, which might be a promising direction to investigate in the context of CAF. Slit2, along with 11 other immune-related genes, was found to have prognostic value for BLCA. Moreover, higher Robo1, the receptor of Slit2, and Slit2 gene expression levels were elevated in advanced stages of BLCA. However, the underlying mechanism of Slit2 in BLCA requires further investigation, which could be facilitated by studying Slit2 in the cell-to-cell communication between CAFs and malignant cells.

Finally, we identified five model genes (SLCO3A1, ANXA1, TENM3, EHBP1, LSAMP) for the construction of a prognostic model, which showed high effectiveness in stratifying patients across seven different cohorts. ANXA1 was identified as one of the 5 genes of prognostic value. A growing body of evidence supports the notion that dysregulations in Anxa1 expression play a critical role in the pathogenesis of cancer, encompassing tumor development, invasion, metastasis, initiation, and acquired resistance to therapies. Recent research suggests that the role of Anxa1 in tumorigenesis is context-dependent, whereby it may act as either a tumor suppressor or a tumor promoter, depending on the specific characteristics of tumor cells or tissues involved. Functional studies have demonstrated that ANXA1 modulated the suppressive function of Treg cells. The latest study on the function of ANXA1 in BLCA has demonstrated that its knockdown prevented PI16’s inhibitory effect on NF-κB activity and cell invasion of BLCA cells, suggesting that ANXA1 might function as a crucial chaperon of PI16 in the control of the NF-κB signaling pathway, and thus participate in the motility and invasion of BLCA cells. Nevertheless, in the research of ANXA1 on drug resistance and relapse in BLCA, a positive correlation was observed between ANXA1 expression levels and the T stage. *In vitro* experiments found that the drug-resistant cell line pumc-91/ADM showed significant downregulation of ANXA1 expression compared to normal pumc-91 cells, suggesting a role of ANXA1 in BLCA drug resistance. Moreover, ANXA1 was shown to foster cell proliferation and migration in BLCA by activating the EGFR signal pathway. Given these findings, ANXA1 holds potential as a valuable biomarker for prognostication in BLCA patients, offering insights into the development of precise and personalized therapeutic strategies for BLCA in the future.

Collectively, we studied the tumor heterogeneity in four cell types, immune cells, endothelial cells, epithelial cells and fibroblasts of BLCA. We delineated the TF activity of the different immune cells in BLCA, highlighting TCF7 and TBX21 enrichment in CD8+ T cells. We divided the CAFs into iCAFs and myoCAFs, which displayed distinct cell communication patterns. The subclusters and cell trajectory analysis showed different states of normal to malignant cell transformation of epithelial cells. TF analysis showed that MYC and SOX2 were highly activated in the tumorous cells. Five model genes, namely SLCO3A1, ANXA1, TENM3, EHBP1, and LSAMP, were screened out for prognostic model construction, displaying high stratification efficacy in seven different BLCA cohorts.

## CONCLUSIONS

Our study discerned the detailed heterogeneity of immune, endothelial, epithelial, and fibroblast cells in BLCA samples. We developed a prognostic model, demonstrating significant efficacy in stratifying BLCA patients.

## References

[1] Zlotta AR, Ballas LK, Niemierko A, Lajkosz K, Kuk C, Miranda G, Drumm M, Mari A, Thio E, Fleshner NE, Kulkarni GS, Jewett MA, Bristow RG, et al. Radical cystectomy versus trimodality therapy for muscle-invasive bladder cancer: a multi-institutional propensity score matched and weighted analysis. Lancet Oncol. 2023; 24:669–81. 10.1016/S1470-2045(23)00170-5 37187202

[2] Patel VG, Oh WK, Galsky MD. Treatment of muscle-invasive and advanced bladder cancer in 2020. CA Cancer J Clin. 2020; 70:404–23. 10.3322/caac.21631 32767764

[3] Tran L, Xiao JF, Agarwal N, Duex JE, Theodorescu D. Advances in bladder cancer biology and therapy. Nat Rev Cancer. 2021; 21:104–21. 10.1038/s41568-020-00313-1 33268841 PMC10112195

[4] Kamat AM, Hahn NM, Efstathiou JA, Lerner SP, Malmström PU, Choi W, Guo CC, Lotan Y, Kassouf W. Bladder cancer. Lancet. 2016; 388:2796–810. 10.1016/S0140-6736(16)30512-8 27345655

[5] Lobo N, Afferi L, Moschini M, Mostafid H, Porten S, Psutka SP, Gupta S, Smith AB, Williams SB, Lotan Y. Epidemiology, Screening, and Prevention of Bladder Cancer. Eur Urol Oncol. 2022; 5:628–39. 10.1016/j.euo.2022.10.003 36333236

[6] Chen H, Wang Z, He Y, Peng L, Zhu J, Zhang X. Pyroptosis may play a crucial role in modifications of the immune microenvironment in periodontitis. J Periodontal Res. 2022; 57:977–90. 10.1111/jre.13035 35839262

[7] Peng L, Chen H, Wang Z, He Y, Zhang X. Identification and validation of a classifier based on hub aging-related genes and aging subtypes correlation with immune microenvironment for periodontitis. Front Immunol. 2022; 13:1042484. 10.3389/fimmu.2022.1042484 36389665 PMC9663931

[8] Chen H, Peng L, Wang Z, He Y, Tang S, Zhang X. Exploration of cross-talk and pyroptosis-related gene signatures and molecular mechanisms between periodontitis and diabetes mellitus via peripheral blood mononuclear cell microarray data analysis. Cytokine. 2022; 159:156014. 10.1016/j.cyto.2022.156014 36084605

[9] Stuart T, Butler A, Hoffman P, Hafemeister C, Papalexi E, Mauck WM 3rd, Hao Y, Stoeckius M, Smibert P, Satija R. Comprehensive Integration of Single-Cell Data. Cell. 2019; 177:1888–902.e21. 10.1016/j.cell.2019.05.031 31178118 PMC6687398

[10] Ma P, Amemiya HM, He LL, Gandhi SJ, Nicol R, Bhattacharyya RP, Smillie CS, Hung DT. Bacterial droplet-based single-cell RNA-seq reveals antibiotic-associated heterogeneous cellular states. Cell. 2023; 186:877–91.e14. 10.1016/j.cell.2023.01.002 36708705 PMC10014032

[11] Simmons SK, Lithwick-Yanai G, Adiconis X, Oberstrass F, Iremadze N, Geiger-Schuller K, Thakore PI, Frangieh CJ, Barad O, Almogy G, Rozenblatt-Rosen O, Regev A, Lipson D, Levin JZ. Mostly natural sequencing-by-synthesis for scRNA-seq using Ultima sequencing. Nat Biotechnol. 2023; 41:204–11. 10.1038/s41587-022-01452-6 36109685 PMC9931582

[12] Kim WJ, Kim EJ, Kim SK, Kim YJ, Ha YS, Jeong P, Kim MJ, Yun SJ, Lee KM, Moon SK, Lee SC, Cha EJ, Bae SC. Predictive value of progression-related gene classifier in primary non-muscle invasive bladder cancer. Mol Cancer. 2010; 9:3. 10.1186/1476-4598-9-3 20059769 PMC2821358

[13] Riester M, Werner L, Bellmunt J, Selvarajah S, Guancial EA, Weir BA, Stack EC, Park RS, O’Brien R, Schutz FA, Choueiri TK, Signoretti S, Lloreta J, et al. Integrative analysis of 1q23.3 copy-number gain in metastatic urothelial carcinoma. Clin Cancer Res. 2014; 20:1873–83. 10.1158/1078-0432.CCR-13-0759 24486590 PMC3975677

[14] Sjödahl G, Lauss M, Lövgren K, Chebil G, Gudjonsson S, Veerla S, Patschan O, Aine M, Fernö M, Ringnér M, Månsson W, Liedberg F, Lindgren D, Höglund M. A molecular taxonomy for urothelial carcinoma. Clin Cancer Res. 2012; 18:3377–86. 10.1158/1078-0432.CCR-12-0077-T 22553347

[15] Choi W, Porten S, Kim S, Willis D, Plimack ER, Hoffman-Censits J, Roth B, Cheng T, Tran M, Lee IL, Melquist J, Bondaruk J, Majewski T, et al. Identification of distinct basal and luminal subtypes of muscle-invasive bladder cancer with different sensitivities to frontline chemotherapy. Cancer Cell. 2014; 25:152–65. 10.1016/j.ccr.2014.01.009 24525232 PMC4011497

[16] Gouin KH 3rd, Ing N, Plummer JT, Rosser CJ, Ben Cheikh B, Oh C, Chen SS, Chan KS, Furuya H, Tourtellotte WG, Knott SR, Theodorescu D. An N-Cadherin 2 expressing epithelial cell subpopulation predicts response to surgery, chemotherapy and immunotherapy in bladder cancer. Nat Commun. 2021; 12:4906. 10.1038/s41467-021-25103-7 34385456 PMC8361097

[17] Powles T, Durán I, van der Heijden MS, Loriot Y, Vogelzang NJ, De Giorgi U, Oudard S, Retz MM, Castellano D, Bamias A, Fléchon A, Gravis G, Hussain S, et al. Atezolizumab versus chemotherapy in patients with platinum-treated locally advanced or metastatic urothelial carcinoma (IMvigor211): a multicentre, open-label, phase 3 randomised controlled trial. Lancet. 2018; 391:748–57. 10.1016/S0140-6736(17)33297-X 29268948

[18] Liu J, Yang TY, Dai LQ, Shi K, Hao Y, Chu BY, Hu DR, Bei ZW, Yuan LP, Pan M, Qian ZY. Intravesical chemotherapy synergize with an immune adjuvant by a thermo-sensitive hydrogel system for bladder cancer. Bioact Mater. 2023; 31:315–332. 10.1016/j.bioactmat.2023.08.013 37663619 PMC10468327

[19] Wang Z, Chen H, Peng L, He Y, Zhang X. Revealing a potential necroptosis-related axis (RP11-138A9.1/hsa-miR-98-5p/ZBP1) in periodontitis by construction of the ceRNA network. J Periodontal Res. 2023; 58:968–85. 10.1111/jre.13157 37357608

[20] Schütz S, Bergsdorf C, Goretzki B, Lingel A, Renatus M, Gossert AD, Jahnke W. The Disordered MAX N-terminus Modulates DNA Binding of the Transcription Factor MYC:MAX. J Mol Biol. 2022; 434:167833. 10.1016/j.jmb.2022.167833 36174765

[21] Bédard M, Maltais L, Montagne M, Lavigne P. Miz-1 and Max compete to engage c-Myc: implication for the mechanism of inhibition of c-Myc transcriptional activity by Miz-1. Proteins. 2017; 85:199–206. 10.1002/prot.25214 27859590

[22] Duffy MJ, O’Grady S, Tang M, Crown J. MYC as a target for cancer treatment. Cancer Treat Rev. 2021; 94:102154. 10.1016/j.ctrv.2021.102154 33524794

[23] Inoue C, Sobue S, Kawamoto Y, Nishizawa Y, Ichihara M, Abe A, Hayakawa F, Suzuki M, Nozawa Y, Murate T. Involvement of MCL1, c-myc, and cyclin D2 protein degradation in ponatinib-induced cytotoxicity against T315I(+) Ph+leukemia cells. Biochem Biophys Res Commun. 2020; 525:1074–80. 10.1016/j.bbrc.2020.02.165 32184020

[24] Anderson DA, Ou F, Kim S, Murphy TL, Murphy KM. Transition from cMyc to L-Myc during dendritic cell development coordinated by rising levels of IRF8. J Exp Med. 2022; 219:e20211483. 10.1084/jem.20211483 34958351 PMC8713298

[25] Nandan D, Rath CT, Reiner NE. Leishmania regulates host macrophage miRNAs expression by engaging transcription factor c-Myc. J Leukoc Biol. 2021; 109:999–1007. 10.1002/JLB.4RU0920-614R 33211335

[26] Gruszka R, Zakrzewski K, Liberski PP, Zakrzewska M. mRNA and miRNA Expression Analyses of the *MYC*/*E2F*/miR-17-92 Network in the Most Common Pediatric Brain Tumors. Int J Mol Sci. 2021; 22:543. 10.3390/ijms22020543 33430425 PMC7827072

[27] Edwards-Hicks J, Su H, Mangolini M, Yoneten KK, Wills J, Rodriguez-Blanco G, Young C, Cho K, Barker H, Muir M, Guerrieri AN, Li XF, White R, et al. MYC sensitises cells to apoptosis by driving energetic demand. Nat Commun. 2022; 13:4674. 10.1038/s41467-022-32368-z 35945217 PMC9363429

[28] Feng YC, Liu XY, Teng L, Ji Q, Wu Y, Li JM, Gao W, Zhang YY, La T, Tabatabaee H, Yan XG, Jamaluddin MF, Zhang D, et al. c-Myc inactivation of p53 through the pan-cancer lncRNA MILIP drives cancer pathogenesis. Nat Commun. 2020; 11:4980. 10.1038/s41467-020-18735-8 33020477 PMC7536215

[29] Wirth M, Stojanovic N, Christian J, Paul MC, Stauber RH, Schmid RM, Häcker G, Krämer OH, Saur D, Schneider G. MYC and EGR1 synergize to trigger tumor cell death by controlling NOXA and BIM transcription upon treatment with the proteasome inhibitor bortezomib. Nucleic Acids Res. 2014; 42:10433–47. 10.1093/nar/gku763 25147211 PMC4176343

